# Digital technology to address HIV and other sexually transmitted infection disparities: Intentions to disclose online personal health records to sex partners among students at a historically Black college

**DOI:** 10.1371/journal.pone.0237648

**Published:** 2020-08-21

**Authors:** Kevon-Mark P. Jackman, Sarah Murray, Lisa Hightow-Weidman, Maria E. Trent, Andrea L. Wirtz, Stefan D. Baral, Jacky M. Jennings

**Affiliations:** 1 Department of Epidemiology, Center for Public Health and Human Rights, Johns Hopkins Bloomberg School of Public Health, Baltimore, Maryland, United States of America; 2 Department of Mental Health, Johns Hopkins Bloomberg School of Public Health, Baltimore, Maryland, United States of America; 3 Institute for Global Health and Infectious Diseases, University of North Carolina at Chapel Hill, Chapel Hill, North Carolina, United States of America; 4 Department of Pediatrics, Adolescent Medicine, Johns Hopkins School of Medicine, Baltimore, Maryland, United States of America; 5 Department of Pediatrics, Center for Child and Community Health Research (CCHR), Johns Hopkins School of Medicine, Baltimore, Maryland, United States of America; Ohio State University, UNITED STATES

## Abstract

Patient portals are creating new opportunities for youth to disclose high-fidelity sexually transmitted infection (STI) laboratory test result histories to sex partners. Among an online survey sample, we describe latent constructs and other variables associated with perceived behavioral intentions to disclose STI test history using patient portals. Participants were co-ed students aged 18 to 25 years (N = 354) attending a southern United States Historically Black College and University in 2015. Three reliable latent constructs were identified by conducting psychometric analyses on 27 survey items. Latent constructs represent, a) STI test disclosure valuation beliefs, b) communication practices, and c) performance expectancy beliefs for disclosing with patient portals. Multivariable logistic regression was used to estimate the relationship of latent constructs to perceived behavioral intentions to disclose STI test history using patient portals. Approximately 14% (48/354) reported patient portal use prior to study and 59% (208/354) endorsed behavioral intentions to use patient portals to disclose STI test history. The latent construct reflecting performance expectancies of patient portals to improve communication and accuracy of disclosed test information was associated with behavioral intentions to disclose STI test histories using patient portals [adjusted odds ratio (AOR) = 1.15; 95% CI = 1.08 to 1.22; *p*<0.001]. Latent constructs representing communication valuation beliefs and practices were not associated with intentions. Self-reporting prior STI diagnosis was also associated with intentions to disclose using patient portals (AOR = 2.84; 95% CI = 1.15 to 6.96; *p* = 0.02). Point of care messages focused on improvements to validating test results, communication, and empowerment, may be an effective strategy to support the adoption of patient portals for STI prevention among populations of college-aged Black youth.

## Introduction

African-American and other Black residents of the United States bear a disproportionate burden of sexually transmitted infections (STIs), including HIV, compared other race and ethnic groups. In 2018, case rates among Black youth aged 15 to 24 years per 100,000 were: 5,085 for chlamydia (versus 1,104 in Whites); 1,793 for gonorrhea (versus 200 in Whites), 49 for primary and secondary syphilis (versus 8 in Whites), and 67 for HIV (versus 6 in Whites) [1,2]. Disclosing accurate STI test histories to sexual partners is a critical component of STI prevention and the Center for Disease Control and Prevention’s “Talk. Test. Treat.” campaign [3–5]. However, several psychosocial and event-level facilitators and barriers can inform the occurrence and accuracy of STI test history disclosures to sex partners among young people [6–8]. When communication exchanges occur, there are variations in language used (e.g., asking “are you good down there?” versus “when was the last time you were tested for HIV?”) and vulnerabilities to inaccurate recall of STI tests performed, test dates, and test results [8–10]. Strategies are needed to reduce communication barriers, improve the fidelity of information exchange, and support healthy normative behaviors around discussing testing with partners among Black youth.

Electronic personal health records (PHRs) are creating new opportunities to increase fidelity and habits around disclosing STI test histories [8,11]. However, little data among Black youth are available on behavioral intentions to adopt patient portals for sharing STI PHRs with sex partners. Patient portals are secure online websites that provide patients with convenient, 24-hour access to their personal health information, such as laboratory test results and prescription medications, referred to as PHRs [12,13]. Patient portals are available as downloadable web-based applications on smartphones, which are widely accessible and used among U.S. populations of Black youth [14–17]. According to Health Information Trends Survey (HINTS) data, a nationally representative survey, 51% of individuals were offered access to their online records in 2018 (versus 42% in 2014); nearly six in 10 of which viewed their PHR at least once [18]. Patient portals are projected to become ubiquitous in healthcare [18,19].

The adoption of patient portals to disclose test history in youth populations can be understood through constructs of behavior theory [20]. Latent constructs are measures of “behaviors, attitudes, and hypothetical scenarios we expect to exist as a result of our theoretical understanding of the world”, assessed using an instrument consisting of survey items or psychometrics [21,22]. Performance expectancy is a construct of technology adoption theory referring to the degree to which the technology provides benefits or relative advantages to executing a task or set of tasks [22,23]. It may be hypothesized that performance expectancies about the interpersonal use of patient portals to disclose STI test history are key determinants of behavioral intentions within populations of Black youth.

According to the Integrative Model of Behavioral Prediction (IMBP), behavioral attitudes, normative beliefs, personal agency, salience of behavior constructs, along with background variables (e.g. biological sex, history of STI infection) determine intentions to perform health behaviors [20,24]. Beliefs about the importance of discussing testing with partners may be anticipated in theory to inform disclosure intentions [25,26]. However, valuation beliefs may vary based on contextual factors, for example, whether condoms are being used. Among youth in Historically Black College and University (HBCU) studies, valuation beliefs among males for soliciting or disclosing STI test history to prospective sex partners hold lower levels of importance compared to female counterparts; slightly smaller proportions of women report sex with partners of unknown HIV status [8,27,28]. History of infection also informs how youth engage in conversations involving test disclosure; for example, by empowerment after receiving chlamydia infection counseling and treatment, or by inhibition related to HIV stigma [29,30]. Identifying the latent constructs and factors relevant to adopting patient portals for disclosing STI test results offers formative data for implementing novel STI prevention strategies.

The goal of this study is to describe perceptions and psychometrics related to using patient portals to disclose test results among an online survey sample of students attending a southern HBCU. Further, to determine whether behavioral intentions to disclose STI test history using patient portals are statistically associated with differences in gender, history of infection, STI test disclosure beliefs, or performance expectancies for disclosing with patient portals.

## Methods

### Study overview

The current study uses online survey data from the Electronic Sexual Health Information Notification and Education (eSHINE) Study. eSHINE (2014–2016) was a two-phase sequential qualitative and quantitative study among co-ed students ages 18–25 years at a southern HBCU exploring perceptions about using patient portals for STI prevention [8,31]. Survey participants were recruited in collaboration with student organizations and university administration to send email blasts advertising the study, table in high-traffic areas, and post study materials across campus spaces. Informed consent was signed in person or online using Adobe EchoSign prior to enrollment. Once consented and enrolled, a secured Qualtrics online survey link was sent to the university email address of each participant. The online survey consisted of 116 items and took an average of 30–45 minutes to complete. Participants were remunerated $20 USD to complete the survey. Study protocols were approved by the Morgan State University Institutional Review Board—(IRB #13/12-0151). eSHINE Study research methods, online survey development, and demographic sample characteristics have been previously described in detail [8].

### Measures

#### Outcome variable

To measure intentions to use PHRs to disclose STI test histories with sex partners, participants were asked to indicate agreement with the statement “I plan to use PHRs in future when discussing STI testing with my partner(s).” Responses ranged from strongly disagree to strongly agree using a 7-point Likert scale. PHRs were defined to participants as “electronic applications that give you electronic access to your medical records (e.g. test results, prescriptions etc.) using your computer, smart phone or tablet.”

#### Other measures

To estimate the proportion of participants with prior PHR experience, participants were asked to indicate (yes/no) whether they have electronically viewed a medical laboratory result. Communication channels were tabulated to provide novel data on the kinds of mass media and interpersonal channels which college-aged Black youth consider important to disseminate messages about using PHRs to disclose STI test history with sex partners. Communication channels refer to sources and characteristics of messages an individual or population receives about adopting new health behaviors, for example, web advertisements (i.e., mass-media sources) or healthcare provider (i.e., interpersonal sources) [32]. Participants were asked to indicate (yes/no) “Who or what would influence your decisions to use PHRs with a partner?” Potential channels emerging from an initial qualitative study included, healthcare providers, sex partners, family, peers, online information, media advertising, and celebrities. Latent constructs representing STI test disclosure beliefs and performance expectancies for disclosing with patient portals were identified using psychometric analysis on a set of 27 survey items described in the following section.

### Statistical analysis

#### Descriptive statistics

Univariate analyses were conducted to describe the study sample by demographic characteristics, sexual risk behaviors, endorsed communication channels, and intentions to disclose.

#### Psychometrics: Latent variable analysis

Exploratory factor analysis (EFA) was used to reduce data and develop reliable latent constructs. First, a principal component analysis (PCA) was performed on 27 survey items measuring a very broad set of communication variables emerging from prior qualitative research, including, (1) beliefs and practices related to STI health communication with sex partners, and (2) performance expectancies related to disclosing test history using PHRs [8]. Item responses used 7-point Likert scales corresponding to scores of -3 to 3. For example: -3 = strongly disagree; -2 = disagree; -1 = somewhat disagree; 0 = neither agree nor disagree; 1 = somewhat agree; 2 = agree; 3 = strongly agree. A complete list of items can be found on S1 Table. Examination of eigenvalues and a parallel analysis were used as the basis for selection of the number of factors to retain in the EFA. In EFA analyses, a minimum factor loading of 0.40 was used as a cut-off for each item [33]. A promax rotation was used, as correlation between factors exceeded 0.32 [34].

Summation of raw scores corresponding to items loading on a specific factor was used to estimate latent construct scores [33]. To measure the internal consistency, Cronbach’s alpha reliability coefficients were calculated overall for each latent construct as determined by the EFA results. To make binary comparisons between, a) participants willing to disclose STI PHRs to partners and b) participants unsure or unwilling to disclose STI PHRs to sex partners, scores of -3 to 0 were categorized as *unsure* or *unwilling* to disclose STI PHRs, and scores of 1 to 3 categorized as *willing* to disclose STI PHRs. Reliability coefficients were calculated by willingness to disclose STI PHRs (willing vs. unsure/unwilling) and by gender (male vs. female). A Cronbach’s alpha value of 0.70 was used as a cut-off value for acceptable internal consistency [35]. Two-sample t-tests were conducted to test differences in mean latent constructs subscale scores by willingness to disclose STI PHRs (willing vs. unsure/unwilling) and by gender at significance *p*<0.05. The Kaiser-Meyer-Olkin (KMO) score for measuring of sampling adequacy for factor analysis was also calculated, with a value of 0.8 or greater considered evidence of adequacy per standard practice [36].

#### Logistic regression analysis

Unadjusted and adjusted multivariable logistic regression models were developed to test associations between emergent latent constructs and background variables on perceived willingness to disclose STI PHRs to sex partners. The perceived willingness variable was categorized as described above, either: a) unsure or unwilling, or b) willing. To build our model, chi-square analyses were conducted on a priori variables anticipated to be associated with willingness to adopt PHR-facilitated STI testing discussions; variables with statistical associations of *p* <0.20 were included in the multivariable model. We then adjusted the models for gender, class standing, and willingness to access STI test results using PHRs (Likert scale variable scores -3 to 3). The latter variable was included in the model since the adoption of STI PHRs is imperative for using such online health services with sex partners. All analyses were conducted using STATA statistical software [37]. Level of statistical significance was pre-defined as *p*< 0.05.

## Results and discussion

### Study population

A total of 1,093 participants registered for the eSHINE Study Online Survey and were sent secured survey links using the university’s student email server. There were 45.8% (501/1,093) who started the survey, of whom 75.8% (380/501) completed the survey and 93.2% (354/380) who completed the survey without missing data. The final analytic sample consisted of 47.2% (167/354) cis-male and 52.8% (187/354) cis-female participants with a median age of 20 years; 96.9% (343/354) identified as Black or African American. Approximately 13.6% (48/354) reported experience viewing an electronic laboratory test result prior to study. Less than half (43.2%; 153/354) reported STI screening in six months prior to the study; 16.7% (59/354) reported a history of STI diagnosis. Messages delivered through healthcare providers (81.4%; 288/354), sex partners (65.2%; 231/354), and family, (54.5%; 193/354) were the most salient communication channels endorsed to influence decisions on adopting the use of HIV/STI PHRs to share test histories (Table 1).

**Table 1:** Demographic characteristics, sexual risk behaviors, and endorsed communication channels believed to influence adoption of patient portals to disclose Sexually Transmitted Infection (STI) test history with sex partners, eSHINE Study Online Survey, 2015 (n = 354).

Variables	Total n (%)
Age	
Median age (IQR)	20 (19–22)
Gender	
Male	167 (47.2)
Female	187 (52.8)
Student Classification	
Freshman	89 (25.1)
Sophomore	82 (23.1)
Junior	87 (25.6)
Senior	88 (24.9)
Graduate student	8 (2.3)
Experience electronically viewing medical laboratory results prior to study	
No	306 (86.4)
Yes	48 (13.6)
Sexual orientation	
Heterosexual	311 (87.9)
Lesbian, gay, or bisexual	43 (12.1)
Reported Sex Partners (in 12 months prior)	
No partners in 12 months prior to study or no history of sexual intercourse	56 (15.8)
1	116 (32.8)
2	79 (22.3)
3–5	78 (22.0)
6+	25 (7.1)
Reported partner-types^a^	
Main partner(s)	213 (60.2)
Casual partner(s)	153 (43.2)
Hook-up partner(s)	72 (20.3)
STI Screening History	
Six months or less prior	153 (43.2)
More than six months prior	81 (22.9)
Never tested	80 (22.6)
No history of sexual intercourse	40 (11.3)
Communication Channels	
Healthcare providers (%yes)	288 (81.4)
Sex partners (%yes)	231 (65.2)
Family members (%yes)	193 (54.5)
Peers (%yes)	169 (47.7)
Online information (%yes)	98 (27.7)
Media advertisements (%yes)	84 (23.7)
Celebrities (%yes)	41 (11.6)

^a^Partner type categories not mutually exclusive.

**Fig 1:**
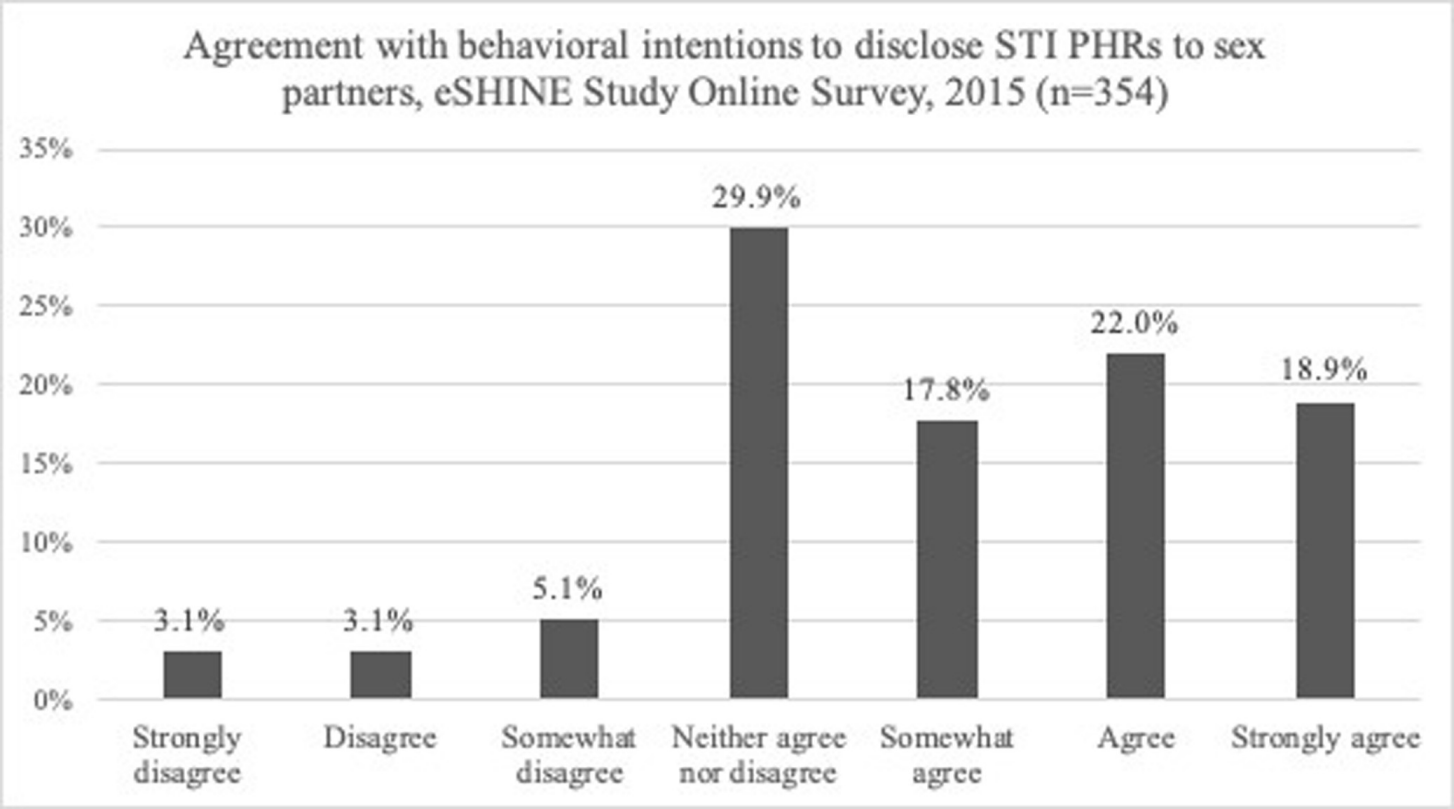
Agreement with behavioral intentions to disclose Sexually Transmitted Infection (STI) electronic Personal Health Records (PHRs) to sexual partners, eSHINE Study Online Survey, 2015 (n = 354).

Fig 1 presents the sample distribution of perceived behavioral intentions to use PHRs for disclosing STI testing histories to sex partners. To summarize, willing participants, scores = 1 to 3, constituted 58.8% (208/354) of the sample. Unwilling participants, scores = -1 to -3, constituted 11.3% (40/354) of the sample. Approximately 29.9% of participants (106/354) neither agreed nor disagreed (score = 0) on intentional beliefs to use PHRs to disclose STI test histories to sex partner.

### Psychometric results

The PCA analysis produced six eigenvalues greater than 1, accounting for 53.3% of the variance. Based on the parallel analysis, we chose a three-factor solution for EFA. The overall KMO score was 0.8231, suggesting the data was adequate for factor analysis. Findings from the 3-factor EFA are presented in Table 2. In total, 16 of the 27 items loaded above the 0.4 thresholds and were retained for the final scale. The remaining 11 items were eliminated from inclusion on any subsequent subscales; no items had cross-loadings above 0.4.

**Table 2:** Factor loadings and uniqueness for exploratory factor analysis using three factor structure and promax rotation conducted on 27 items measuring beliefs and practices related to sexually transmitted infection health communication with sex partners, eSHINE Study Online Survey, 2015 (n = 354).

Variable	Factor loadings^a^		
	Factor 1. Communication Valuation	Factor 2. Communication Practice	Factor 3. PHR Impact
1. How will PHRs^b^ affect: Control over my sexual health and decision making.	0.0088	-0.0302	**0.7675**
2. How will PHRs affect: Confidence in the testing information a partner shares with me	0.0552	-0.0447	**0.8031**
3. How will PHRs affect: Communication between my partner(s) and myself	0.0669	-0.0144	**0.8008**
4. PHRs make it easier for people to routinely have "check in" conversations with partners about STI prevention	-0.0142	-0.0164	**0.6835**
5. Partners using PHRs will start talking about STI prevention EARLIER in a relationship	-0.0639	0.0238	**0.6335**
6. I would have more discussions with partners about STI testing if PHRs were more commonly used.	-0.0294	-0.0126	**0.5405**
7. Using PHRs with a partner builds trust	-0.1038	0.0680	**0.6454**
8. PHRs make it easier to discuss STI testing when intoxicated	0.0175	-0.0927	0.3178
9. Asking partner(s) to view their electronic STI results will make things awkward.	0.3111	0.0268	0.0330
10. How likely would it upset you if your partner asks to see your PHR after you have told them your STI testing results.	0.3696	-0.1464	-0.0774
11. My partner(s) and I would not want to use PHRs for risk discussions because we trust each other.	0.3183	-0.0792	0.1066
12. I will be suspicious if a partner is unwilling to share their electronic STI results with me	0.1066	0.0479	0.3810
13. Partners that have been drinking alcohol or using other drugs are LESS likely to use condoms when their electronic STI records show NO infections.	-0.1530	0.0861	0.3481
14. Discussing STI testing with my partner(s) demonstrates that I care about my health.	0.3795	-0.0750	0.1042
15. People have the right to ask partner(s) information about their STD testing.	0.3709	0.0792	0.1039
16. How important is it for you to discuss STI testing with new or potential sexual partners?	**0.6789**	-0.0678	0.0162
17. How important is it for you to know information about your partner’s most recent STI test?	**0.5979**	0.0178	-0.0281
18. How important is it for you to know information about your partner’s condom use with previous partner(s)?	**0.5312**	0.1305	-0.0084
19. If my partner(s) and I decide to use condoms, how important is it for us to discuss STI testing?	**0.6392**	0.1082	-0.0954
20. If my partner(s) and I decide to use condoms, how important is it for us to discuss our electronic STI results?	**0.5627**	-0.0072	0.1869
21. How likely are you to ask a new or potential sex partner: "How many people have you had sex with?"	-0.0273	**0.7336**	-0.0306
22. How likely are you to ask a new or potential sex partner: "Do you have any STIs or HIV"?	0.1939	**0.4750**	0.0063
23. How likely are you to ask a new or potential sex partner: "Are you good down there?" or "Are you clean?"	0.0866	**0.5197**	-0.0369
24. How likely are you to ask a new or potential sex partner: "Who were you having sex with before me?"	0.0202	**0.7246**	-0.0182
25. How likely are you to ask a partner to see their electronic STI results if you think they may be offended?	0.2570	-0.0300	0.2112
26. I would not use a condom if my partner's most recent electronic STI results are negative (i.e. they are clean)?	-0.2723	0.1091	0.1866
27. How easy or difficult is it to have risk discussions about STI testing with your partner(s)?	0.2866	0.1865	0.0191

^a^Items loading above the .40 threshold are in **bold font**.

^b^PHRs: electronic personal health records.

Based on factor loadings, three latent constructs were identified, *communication valuation*, *communication practice*, and *PHR impact* (Table 2). PHR impact had seven items load over 0.4; these items reflect the performance expectancies of using STI PHRs in communication with partners. The highest loading items centered on attributes of improved health communication between sexual partners, assurance in shared screening information, and control over sexual health and decision making. Communication valuation had five items load over 0.4; items assess the perceived importance of discussing STI testing with sexual partners. Communication practice had four items load over 0.4; items represent the thoroughness of STI risk information solicited from sexual partners, e.g., likelihood to solicit information about prior sex partners, number of lifetime partners, or STI status disclosure.

Table 3 shows the Cronbach's alpha reliability coefficients and mean scores for latent construct, and additionally disaggregated by behavioral intentions to use PHRs for disclosing STI test history and by gender. Reliability coefficients ranged from 0.74–0.86 with the PHR impact subscale having the highest internal consistency. Reliability coefficients in bivariate comparisons were lowest (0.69) for the communication practice latent construct. Compared to male participants, scores for communication valuation (mean = 11.62 vs. mean = 9.70; t = 4.58; *p*<0.001) and communication practice (mean = 8.04 vs. mean = 6.15; t = 3.40; *p* = 0.001) were significantly higher among female participants.

**Table 3:** Cronbach’s alpha (α), mean score (x¯), and standard deviation (SD) values for emergent exploratory factor analysis (EFA) latent constructs of sexually transmitted infection (STI) testing communication with sexual partners, by gender and by willingness to use patient portals to disclose STI test history with partners, eSHINE Study Online Survey, 2015 (n = 354).

Variable	Latent constructs^a^					
	F1. Dyadic Communication Valuation		F2. Dyadic Communication Practice		F3. PHR Impact	
	α	x¯ (SD)	α	x¯ (SD)	α	x¯ (SD)
**Total Sample** (n = 354)	0.76	10.72 (4.06)	0.74	7.15 (5.30)	0.85	10.83 (6.70)
Gender						
Male (n = 167)	0.71	9.70 (4.31)	0.77	6.15 (5.70)	0.87	11.16 (6.69)
Female (n = 187)	0.79	11.62 (3.58)	0.69	8.04 (4.74)	0.85	10.54 (6.72)
*|t| (df); P-value*^b^	*4*.*58 (352); P<0*.*001*		*3*.*40 (352); P = 0*.*001*		*0*.*88 (352); P = 0*.*39*	
Intentions to disclose STI PHRs^c^ to partners (*dichotomized*)						
Unsure/ Unwilling (n = 146)	0.78	10.17 (4.28)	0.80	6.82 (5.60)	0.83	6.92 (6.63)
Willing (n = 208)	0.73	11.10 (3.85)	0.69	7.38 (5.07)	0.78	13.58 (5.23)
*|t| (df); P-value*	*2*.*14 (352); P = 0*.*03*		*0*.*98 (352); P = 0*.*33*		*10*.*55 (352); P<0*.*001*	

^a^Interscale correlations: r_f1, f2_ = 0.37, *P* < .001; r_f1, f3_ = 0.23, *P* < .001; r_f2,f3_ = 0.11, *P* = .04.

^b^Two-sample t-test whether observations significantly differ by group; |t value|, degrees of freedom (df); and P-value are presented.

### Unadjusted and adjusted multivariable logistic regression

Table 4 shows odds ratios for unadjusted and adjusted multivariable logistic regression models. In the unadjusted model, PHR impact and communication valuation are both significantly associated with intentions to share STI test histories with PHRs. When adjusted for gender, student classification, screening history, history of STI diagnosis, and emergent latent factors; only PHR impact remains as a significant factor predicting willingness to adopt PHRs to share STI test history [adjusted odds ratio (AOR) = 1.15; 95% CI = 1.08 to 1.22; *p*<0.001]. Neither communication valuation nor communication practice were significantly associated with perceived behavioral intentions for STI PHR disclosure.

**Table 4:** Unadjusted and adjusted multivariable logistic regression on willingness to disclose Sexually Transmitted Infection (STI) online Personal Health Records (PHRs) to sexual partners, eSHINE Study Online Survey, 2015 (n = 354).

Predictors		Logistic Regression Models^a^			
		Unadjusted odds ratio (95% CI)	*P*-value	Adjusted odds ratio (95% CI)	*P*-value
Gender					
	Male	ref		ref	
	Female	0.80 (0.52, 1.22)	0.29	0.81 (0.43, 1.54)	0.52
Class standing					
	Freshman	ref		ref	
	Sophomore	**0.51 (0.27, 0.94)**	**0.03**	**0.34 (0.14, 0.79)**	**0.01**
	Junior	**0.54 (.029, 1.00)**	**0.05**	**0.36 (0.15, 0.84)**	**0.02**
	Senior	0.81 (0.43, 1.49)	0.49	0.76 (0.31, 1.82)	0.53
	Graduate student	0.81 (0.18, 3.60)	0.78	0.82 (0.14, 4.93)	0.82
Most recent STI test					
	6 months	ref		ref	
	≥ 7 months	0.65 (0.37, 1.12)	0.12	**0.43 (0.20, 0.94)**	**0.04**
	Never tested	**0.57 (0.33, 0.99)**	**0.05**	0.58 (0.26, 1.29)	0.18
	No exposure	0.82 (0.40, 1.67)	0.58	1.28 (0.46, 3.55)	0.64
History of HIV/STI diagnosis					
No		ref		ref	
Yes		**2.12 (1.14, 3.93)**	**0.02**	**2.84 (1.15, 6.96)**	**0.02**
Willingness to adopt the use of STI PHRs^b^		**3.36 (2.56, 4.40)**	**<0.001**	**2.96 (2.19, 4.01)**	**<0.001**
STI Health Communication Subscale					
	Communication Valuation	**1.05 (1.00, 1.11)**	**0.04**	0.96 (0.89, 1.05)	0.40
	Communication Practice	1.02 (0.98, 1.06)	0.33	1.01 (0.95, 1.08)	0.75
	PHR Impact	**1.21 (1.16, 1.27)**	**<0.001**	**1.15 (1.08, 1.22)**	**<0.001**

^a^**Bold font** used to emphasize statistical significance, *p*<0.05.

^b^PHRs: electronic personal health records.

Participants reporting a history of prior STI diagnosis were significantly more likely to support sharing STI PHRs with partners (AOR = 2.84; 95% CI = 1.15 to 6.96; *p* = .02). Additionally, compared to participants reporting recent STI screening, reporting STI screening more than six months prior to the study was significantly associated with lower odds of adoption (AOR = 0.43; 95% CI = 0.20 to 0.94; *p* = .04). Finally, compared to freshman students, sophomore and junior students were significantly less willing to use HIV/STI PHRs for sharing test histories [(AOR = 0.34; 95% CI = 0.14 to 0.79; *p* = .01) and (AOR = 0.36; 95% CI = 0.15 to 0.84; *p* = .02), respectively].

### Principal findings

With the goal of offering formative data to research focused on empowering populations of Black youth to disclose high-fidelity STI test information to sex partners, we identified the latent constructs and background variables relevant adopting patient portals for STI test disclosure among a sample of students attending a HBCU. Overall, most participants are willing to use the PHRs within patient portals to facilitate conversations with their partners on STI testing. We identified three latent constructs representing psychometric domains of STI test disclosure communication between partners. Latent construct factors had good internal consistency with reliability coefficients ranging from 0.74–0.86 overall with similar findings when stratified by intentions to adopt PHRs for STI test disclosure and by gender. Intentions to use PHRs to disclose STI test histories was significantly associated in adjusted multivariable analyses with class standing, screening history, history of STI diagnosis, intentions to use STI PHRs, and the PHR impact latent construct.

Findings add to scientific literature on the acceptability of adopting online health technology to foster engagement with sexual health care and communication with sexual partners among youth [38–40]. Further, it builds upon our prior work dissecting motivations and norms around discussing STI testing and disclosure with partners [8]. Although moderately correlated, communication valuation and communication practice subscales are distinct and help delineate between sometimes-contradicting dynamics between behavioral attitudes and personal agency when constraining conditions are present [20]. Gender differences in communication valuation and communication practice scores supports research suggesting that facilitating conversations may be more important to young Black women compared to men in a largely heterosexual context [27,28]. Nevertheless, there are no significant gender differences with respect to perceptions about using PHRs for disclosure. In fact, male participants had higher PHR impact scores and were more willing to adopt use PHRs in communication with sex partners–however, these differences were not significant. Patient portals may be a promising vehicle to deliver tailored interventions to uniquely address gender-based risk patterns for STI among youth [41,42].

The role of PHRs as a private, convenient, and easy to use sexual health management tool that supports sexual health awareness may have upstream effects on decisions to use STI PHRs in disclosures [23,31,43]. Trust in privacy and security may be particularly important for youth with a history of STI diagnosis. The significant association between prior STI diagnosis and willingness to adopt PHRs for disclosure is supportive qualitative findings where participants described that the experience of receiving a STI diagnosis increased the importance of discussing testing with future partners [8]. Further studies are needed to determine how patient portals may help sero-discordant sexual partners in navigating complexities of discussing prevention and care; particularly for chronic infections, such as HIV and herpes simplex virus type-2 [44].

Clinicians and allied health professionals may be key influencers of how youth adopt the use of patient portals for STI test disclosures as new health behaviors. The PHR impact latent construct may be collected (i.e., using an electronic health record (EHR) e-form) in clinical settings to prioritize the delivery interventions empowering STI test disclosure [45,46]. Sex partners and family may additionally be effective interpersonal communication channels to support the adoption of sharing STI PHRs. Similarly, patient portals may also include modules to with role plays for how to discuss testing with sex partners. Still, mass media communication channels are initially important to broadly spreading awareness about new innovations [32]. Messages should focus on beneficial innovation attributes, particularly improvements to validating test results, communication, and empowerment. In college settings, freshman students may be more receptive to dyadic STI PHR use. Freshman orientations may provide an opportunity for promoting such interventions.

### Limitations

There are several limitations to our study. Reported intentions to share STI PHRs may be biased where participants with attitudes opposed to STI test disclosures perceive support for disclosure as a more socially acceptable survey response, referred to as social desirability bias [47]. Limited real-life experiences accessing STI PHRs in the sample may have contributed to the large number of participants undecided about using patient portals in disclosures. Deciding on intentions may also be difficult without explicating the myriad contextual factors that influence individual-level attitudes and practices related to STI test disclosure between sex partners [8]. Extrapolation of our findings are limited by the study population and convenience sampling. Future studies are needed to explore behavioral intentions among Black and other minority adolescents and young adults with less than or equivalent to a high school education.

### Conclusions

Getting youth to talk with sex partners about testing and healthy sexual behaviors remains a public health challenge and a critical component of the “Talk. Test. Treat.” campaign [5,6]. Adding a STI prevention infrastructure and capacity-building lens to the implementation of patient portals offers new strategies for addressing longstanding racial disparities. Such interventions may focus on reducing the stigma around STI health communication among youth, their sex partners, and their health care providers [6,48–50]. However, the success of future interventions requires public health priorities focused on patient portal access to STI PHRs and incentives to design patient portal platforms to support sexual and reproductive health among Black youth.

## Supporting information

S1 Table Items included in exploratory factor analysis, willingness to adopt PHR delivered results and willingness to adopt PHR facilitated risk discussions–eSHINE Study Online Survey (2015). (DOCX)

S1 Dataset eSHINE study dataset file. (DTA)
