# Supplementary material for: Digital technology to address HIV and other sexually transmitted infection disparities: Intentions to disclose online personal health records to sex partners among students at a historically Black college
Source: PLoS One. 2020 Aug 21;15(8):e0237648. doi: 10.1371/journal.pone.0237648 (PMC7442257; doi:10.1371/journal.pone.0237648)
Supplement: S1 Table — (DOCX) [file pone.0237648.s001.docx]

| **S1 Table. Items included in exploratory factor analysis, willingness to adopt PHR delivered results and willingness to adopt PHR facilitated risk discussions – eSHINE Study Online Survey (2015)** | |
| --- | --- |
| **Online Survey Items** | **Answer Options** |
| PA_SC1. How will PHRs affect: Control over my sexual health and decision making.  PA_SC2. How will PHRs affect: Confidence in the testing information a partner shares with me  PA_SC3. How will PHRs affect: Communication between my partner(s) and myself | a. Very harmful  b. Harmful  c. Somewhat harmful  d. Neutral  e. Somewhat helpful  f. Helpful  g. Very Helpful |
| PA_SC4. PHRs make it easier for people to routinely have "check in" conversations with partners about STI prevention  PA_SC5. Partners using PHRs will start talking about STI prevention EARLIER in a relationship  PA_SC6. I would have more discussions with partners about STI testing if PHRs were more commonly used.  PA_SC7. Using PHRs with a partner builds trust | a. Strongly disagree  b. Disagree  c. Somewhat disagree  d. Neither agree nor disagree  e. Somewhat agree  f. Agree  g. Strongly Agree |
| PA_SC8. PHRs make it easier to discuss STI testing when intoxicated  PA_SC9. Asking partner(s) to view their electronic STI results will make things awkward.  PA_SC10. How likely would it upset you if your partner asks to see your PHR after you have told them your STI testing results.  PA_SC11. My partner(s) and I would not want to use PHRs for risk discussions because we trust each other.  PA_SC12. I will be suspicious if a partner is unwilling to share their electronic STI results with me  PA_SC13. Partners that have been drinking alcohol or using other drugs are LESS likely to use condoms when their electronic STI records show NO infections.  PA_SC14. Discussing STI testing with my partner(s) demonstrates that I care about my health.  PA_SC15. People have the right to ask partner(s) information about their STD testing. | a. Strongly disagree  b. Disagree  c. Somewhat disagree  d. Neither agree nor disagree  e. Somewhat agree  f. Agree  g. Strongly Agree |
| PA_SC16. How important is it for you to discuss STI testing with new or potential sexual partners?  PA_SC17. The next question asks how important certain information is for you to know about your sex partner(s)?: Information about their most recent STI test  PA_SC18. The next question asks how important certain information is for you to know about your sex partner(s)?: Information about their condom use with previous partner(s).  PA_SC19. If my partner(s) and I decide to use condoms, how important is it for us to discuss STI testing?  PA_SC20. If my partner(s) and I decide to use condoms, how important is it for us to discuss our electronic STI results? | a. Not at all important  b. Very unimportant  c. Somewhat unimportant  d. Neither important nor unimportant  e. Somewhat important  f. Very important  g. Extremely important |
| PA_SC21. How likely are you to ask a new or potential sex partner: "How many people have you had sex with?"  PA_SC22. How likely are you to ask a new or potential sex partner: "Do you have any STIs or HIV"?  PA_SC23. How likely are you to ask a new or potential sex partner: "Are you good down there?" or "Are you clean?"  PA_SC24. How likely are you to ask a new or potential sex partner: "Who were you having sex with before me?"  PA_SC25. How likely are you to ask a partner to see their electronic STI results if you think they may be offended? | a. Very unlikely  b. Unlikely  c. Somewhat unlikely  d. Neutral  e. Somewhat likely  f. Likely  g. Very likely |
| PA_SC26. I would not use a condom if my partner's most recent electronic STI results are negative (i.e. they are clean)? | a. Strongly disagree  b. Disagree  c. Somewhat disagree  d. Neither agree nor disagree  e. Somewhat agree  f. Agree  g. Strongly Agree |
| PA_SC27. How easy or difficult is it to have risk discussions about STI testing with your partner(s)? | a. Very difficult  b. Difficult  c. Somewhat difficult  d. Neutral  e. Somewhat easy  f. Easy  g. Very easy |
